# Health Food Company

**Published:** 1875-09

**Authors:** 

**Affiliations:** 137 Eighth St., near Broadway, N. Y.


### THE HEALTH FOOD COMPANY.

We cheerfully give place to a communication written by the worthy President of the Health Food Company, which contains valuable thoughts, although perhaps a little too complimentary to the JOURNAL and its influence ;

*To the Editor of Hall's Journal of Health :*

The very kindly interest which you have manifested in our efforts to introduce better foods to the notice of the world, and the great impetus which your intelligent comments have given to our work, make appropriate some words of grateful thanks. When we, years ago, conceived the idea of preparing health foods for public consumption, the enthusiastic little circle of friends, pledging themselves to the work, unanimously agreed that the JOURNAL should be the first advertising medium employed. This decision arose from the fact that the course of your magazine had always been reasonable and consistent, and that its teachings had uniformly been to the effect that good food is better than medicine.

As soon as the period for experimenting was ended and we had got our costly machinery in complete working condition ; as soon as we thought the product of our ovens had reached the absolute perfection for which we had labored fully ten years,—we submitted to you our Granulated Wheat Biscuit, described our processes and machinery, and arranged for the insertion of our advertisement. That advertisement you kindly accompanied with a very clear and interesting editorial notice, in which you earnestly recommended our new food. This was only two months ago.

The result of this publicity has been to secure for our food a demand which we supposed it would require a year or two to build up. As we have advertised in no other publication we are able to give the credit where it is solely due—to your excellent JOURNAL OF HEALTH. For what you have so freely and so intelligently said of us and of our food, we thank you heartily.

You have forced us to enlarge our works, and in a month or so, our productive power will be more than doubled. The new machinery will give us a capacity of fully one ton daily, and with our new oven,—three stories high, each soap-stone floor having a surface of 900 square feet, or 2700 square feet of baking surface in all—we hope to supply all demands.

We have learned that no more grateful article of food for some peculiarly irritable and weak stomachs, can be found. For children and invalids, it may be soaked in new milk, and the milk may be drained off and swallowed with the most satisfactory results. The delicate flavor of toast imparted to the milk, makes it very palatable and very agreeable to the stomach, and the nutriment extracted from the biscuit renders it a very strengthening diet. Some dyspeptics who cannot eat milk without suffering, are able to partake abundantly of milk in which this granulated food has been soaked.

The biscuits are necessarily a somewhat costly food. They probably actually cost one-third more than the best water-crackers in the market. The wheat is the best grown, and the processes are careful and long-continued, yet the price is less than that of some of the brands of crackers, while experience proves that the granulated biscuit contains not less than twice as much actual food as any crackers made. Thus, if they were sold at double the present price, they would be cheaper than any other food sold.

In justice to your greatly valued commendation of this new food, we would assure you that the standard of excellence will be fully sustained, and that the biscuit will be improved if improvement is found possible.

Nearly all the orders for the biscuits come by mail and usually enclose money to pay for the amount ordered, at the rate of

15 cents per pound. To this price, an amount sufficient to pay postage at the rate of 16 cents per pound, should be added, if a few pounds are desired by mail. Some have written on postal cards, asking that a sample be sent by mail. We have tried to accommodate all such correspondents, but the burden has been considerable. It is not merely the cost of the biscuit, but the added expense of postage which makes this labor a trifle onerous. So we have decided to exact a three-cent stamp from all to whom we send samples by mail. For this we shall mail two ounces of the biscuits, the postage on which will be two cents. The remaining cent will do something towards the cost of the two ounces of food sent, and the envelope covering it.

Ladies and gentlemen calling at our place of business can sample the biscuit at all times freely and to their hearts content.

Yours truly,

THE HEALTH FOOD CO.,

137 Eighth St., near Broadway, N. Y.

